# Creating Compassionate Spaces for End-of-Life Care for Older People Experiencing Homelessness: Protocol for an Environmental Assessment of Hospice Settings

**DOI:** 10.2196/73356

**Published:** 2025-11-05

**Authors:** Atiya Mahmood, Sally Seohyeon Chung, Nushaiba Nanjiba, Sepehr Pandsheno, Jeffrey J Walsh, Sharmin Kader, Sarah L Canham

**Affiliations:** 1 Department of Gerontology Simon Fraser University Burnaby, BC Canada; 2 McGill University Montreal, QC Canada; 3 Department of Construction Management and Interior Design Ball State University Muncie, IN United States; 4 College of Social Work University of Utah Salt Lake City, UT United States

**Keywords:** end-of-life, homelessness, hospice, older adults, protocol

## Abstract

**Background:**

With current data supporting an increasing population of older people experiencing homelessness (OPEH) requiring unique spatial and placemaking considerations in end-of-life care, understanding the environmental factors that influence their well-being is crucial.

**Objective:**

This protocol paper provides a comprehensive overview for evaluating hospice environments tailored to the needs of OPEH.

**Methods:**

The Aging in the Right Place study aims to address this gap by developing and implementing the Aging in the Right Place-Hospice Environmental Assessment Protocol (AIRP-HEAP) and AIRP-HEAP secondary observation tools. The AIRP-HEAP tool evaluates the built and natural environment within hospice settings. Adaptations were made to ensure alignment with the unique needs of OPEH, such as reconceptualizing spiritual care and expanding the definition of family accommodation. Additionally, the AIRP-HEAP secondary observation tool supplements this by capturing contextual data on the surrounding neighborhood of the hospice site, providing a holistic understanding.

**Results:**

Data were collected at Maggie’s Lodge hospice between November and December 2024 using the AIRP-HEAP and AIRP-HEAP secondary observation tools. The dataset is currently being cleaned, with analysis planned between May and December 2025. The anticipated results will highlight the importance of considering environmental factors in hospice environments and inform recommendations to improve end-of-life care for OPEH.

**Conclusions:**

Data collected using these audit tools can guide environmental modifications in hospice settings to facilitate aging and end-of-life care in the right place. Thus, this protocol paper aims to promote the adoption of best practices in hospice design to better support this marginalized population.

**International Registered Report Identifier (IRRID):**

DERR1-10.2196/73356

## Introduction

Homelessness is a growing social problem in Canada, especially among older adults. According to a point-in-time survey, which provides a glimpse into the minimum number of people experiencing homelessness in a 24-hour period, 4821 people were experiencing homelessness in Vancouver [1]. This number marks a stark 32% increase from the point-in-time survey conducted in 2020 [1]. The proportion of older people experiencing homelessness (OPEH) has also risen, comprising 22% of the homeless population [2]. Safe and stable housing is instrumental in facilitating the physical, mental, and emotional well-being of older adults [3,4]. As the homeless population ages, and as the aging population experiences greater housing insecurity, their demands on medical services, home care, and end-of-life care increase [5]. Research on the Vancouver homeless population reveals the population experiences higher rates of premature death and physical illness [6], while broader research on homelessness demonstrates increased rates of cancer [7] and chronic illnesses [8] compared to the housed population. Moreover, individuals experiencing homelessness often require access to health care services 10-20 years earlier than the housed population due to frailty and age-related conditions [9].

Despite an increased need for palliative care, OPEH face barriers not experienced by the general population. The built environment has been demonstrated to include or exclude OPEH from palliative care. For example, many community-based palliative care programs often deny care to OPEH because the services deem them as not having a physical space to provide medical care [10,11], or because the buildings or neighborhoods they live in are deemed unsafe [11,12]. The built environment also facilitates or hinders OPEH access to institutional palliative care programs. Physical and aesthetic features of these built environments signal to OPEH their acceptance or rejection into the space [13]. Similarly, institutional environments mirroring Indian residential schools or Indian hospitals indicate to Indigenous people that the space is unsafe or traumatic [12,13]. The built environment plays a key role in meeting the end-of-life needs of OPEH.

The concept of “end-of-life care in the right place” emphasizes the importance of providing appropriate housing that aligns with individuals’ unique needs and lifestyles as they approach the end of their lives. This concept holds particular importance for OPEH, a demographic whose palliative needs are shaped by experiences of marginalization, colonialism, and trauma [14]. Conceptualizations of key palliative care tenets, such as safety and autonomy, are shaped differently within this community. Discrimination and abuse experienced when accessing health care greatly influence the meaning of safety within a health institution [13,14]. Similarly, higher rates of substance use and mental illness translate into unique spatial and placemaking needs for OPEH at end-of-life [12,13].

Within the Canadian landscape of end-of-life care, hospice care plays an essential role. The term “hospice” can refer to either a philosophy of care or a physical care facility providing end-of-life care to people. The former definition expresses a philosophy of care that offers medical, social, and emotional support at the end of life, which can be provided at home or within a care facility [15]. The latter definition represents an integral space-based approach where a hospice philosophy is used to address the end-of-life care needs of individuals within a purpose-built facility. Given the barriers to palliative care due to the built environment, specialized hospice facilities for OPEH hold the potential to address this population’s end-of-life care needs.

The spatial understanding of hospices fits with the historical development of the concept, dating back to physical buildings that provided care to sick and dying individuals [16]. Understanding hospices as physical facilities for end-of-life care allows us to examine how their physical make-up either facilitates or inhibits effective care for individuals at the end of life. This understanding is crucial for enhancing our knowledge of how the built environment influences end-of-life care in the right place. For OPEH, the built environment of hospice facilities is especially significant, as research underscores the critical role that space plays in their access to end-of-life care [10,17]. Environmental audits (also referred to as environmental observations or assessments) are useful in evaluating the built environment and can identify ways to improve the environment and placemaking characteristics in different types of housing, including hospice settings. Canham and colleagues [3] reported findings from an environmental audit of a temporary housing program for OPEH, which highlighted the integral role of spatial and placemaking aspects to aging in the right place. In this study, we extend this previous research by applying a therapeutic goals lens [18] to evaluate the extent to which spatial and placemaking factors (eg, spatial configuration, design and layout, connections to nature, access, and scope of personalization) can promote comfort, privacy, sociability, control, dignity, and spiritual expression in hospice setting to understand whether these settings are appropriate for aging and end-of-life care in the right place for OPEH. To examine the built environment of this specialized hospice care (Maggie’s Lodge), we developed an Aging in the Right Place-Hospice Environmental Assessment Protocol (AIRP-HEAP) tool.

End-of-life in the right place is an extension of the Aging in the Right Place (AIRP) project, which emphasizes the significance of secure and optimal housing that assists an individual’s unique vulnerabilities [12]. The concept of AIRP acknowledges that what constitutes the right place for an older adult to age will vary from person to person [19]. Canham et al [3] devised an AIRP project framework drawing on insights from experts in aging, homelessness, and housing services, as well as older adults with lived experience of homelessness. The AIRP project’s framework comprises 6 subcategories, which are indicators for aging in the right place for OPEH. These subcategories are (1) built environment of the housing unit and surrounding neighborhoods, (2) off-site and on-site health and social services and resources, (3) social integration, (4) stability and affordability of space, (5) emotional place attachment, and (6) broader political and economic context. This protocol paper’s focus is on the built environment audit, thus the first indicator of the framework, which is the built environment of the housing unit and the surrounding neighborhoods. An unsupportive built environment can have damaging effects on the quality of life of OPEH. Given that this paper is based on the development of an environment audit tool, the built environment identifies notable features of the building, such as amenity rooms, green spaces, security cameras, etc [3]. The AIRP project documents and evaluates “promising practices” of shelter and housing that support aging in the right place for OPEH. Promising practices are understood as novel models that have not gone through rigorous evaluation but hold the promise of supporting aging in the right place for OPEH [18]. One such promising practice for the Vancouver site of the AIRP project is Maggie’s Lodge, a 6-bed hospice with a home-like setting and 24-hour nursing care embedded in a socioeconomically marginalized neighborhood in Vancouver, and the only hospice in British Columbia (and one of a select few in Canada) with a mandate to provide end-of-life care for OPEH [18].

## Methods

### Audit Tool Development

The AIRP-HEAP tool is an adapted version of the Hospice Environmental Assessment Protocol (HEAP) tool developed by Kader [20] as part of her doctoral dissertation. The HEAP tool was developed following the conceptual framework of the Professional Environmental Assessment Protocol (PEAP), a widely recognized, evidence-based tool for evaluating the quality and therapeutic potential of long-term care and dementia care environments [21,22]. The HEAP tool was developed as a standardized method for evaluating whether the spatial and placemaking characteristics of hospice buildings support the following 11 therapeutic goals: (1) continuity of self, (2) access to nature, (3) privacy, (4) social interaction, (5) safety and security, (6) autonomy, (7) stimulation and sensory therapies, (8) spiritual care, (9) family accommodation, (10) support after death, and (11) support for staff [20] (see Textbox 1 for definitions of each goal). Each goal includes several design objectives and subobjectives that operationalize the respective goal, and each objective includes specific environmental items that are observable within the hospice environment, totaling 295 items. For example, the therapeutic goal of “continuity of self” has the following two design objectives: (1) creating a noninstitutional environment or home-like environment and (2) scope for personalization, which each have specific environmental items. The “scope for personalization” objective includes wall shelves present to display personal belongings, and adequate space in individual rooms is provided to bring personal belongings. We adapted these 11 therapeutic goals for the AIRP-HEAP tool to be conceptually linked to domains within the AIRP project’s framework [3]. The AIRP project’s framework was developed to provide a practical conceptualization of 6 indicators that contribute to aging in the right place for OPEH, and the AIRP-HEAP tool domain was based on this framework [3]. Based on this framework, the AIRP-HEAP tool domains are (1) built and natural environment; (2) housing access and home modification; (3) emotional place attachment; (4) social support, participation, and inclusion; and (5) safety and security. The synergy between the AIRP framework’s concepts and the therapeutic goals of the AIRP-HEAP tool helps to ground the environmental characteristics of hospice sites within the conceptual discussion of aging and end-of-life care in the right place. Each audit domain is intentionally structured to theoretically align with the AIRP framework, as illustrated in Textbox 2. This bridging of theory and measurement allows the audit to focus on the environmental elements that support the distinct needs and vulnerabilities of older adults in the hospice setting.

**Eleven therapeutic goals of the Hospice Environmental Assessment Protocol tool.**

**Provide continuity of self**

- Environmental characteristics that help preserve or support patients’ past activities, preferences, and awareness

**Provide access to nature**

- Environmental characteristics that provide opportunities for visual and physical access to nature

**Provide privacy**

- Environmental characteristics that facilitate patients’ choices in various levels of privacy through regulation of visual and auditory stimuli

**Facilitate social interaction**

- Environmental characteristics that facilitate and enable meaningful interaction between patients with staff, their family, and other patients

**Maximize safety and security**

- Environmental characteristics that maximize safety and security of patients, staff, and families

**Provide autonomy**

- Environmental characteristics that enable patients to exercise choice and personal preference about their environment and everyday life

**Regulate stimulation and support sensory therapies**

- Environmental characteristics that contribute to an appropriate quantity and quality of sensory experience, and support sensory therapies (palliative therapies)

**Provide spiritual care**

- Environmental characteristics that facilitate opportunities for patients’ spiritual care; religious, philosophical, and existential or personal beliefs, values, practices, and preferences

**Provide family accommodation**

- Environmental characteristics that facilitate patients’ family accommodation and support control, functional independence, comfort, privacy, recreation, and spiritual care

**Provide support after death**

- Environmental characteristics that support care and dignity for patients and their families during the moment of death, body removal, bereavement, and remembrance

**Maximize support for staff**

- Environmental characteristics that support staff (care providers) for better communication, observation, efficiency, satisfaction, and well-being

**Link between the Aging in the Right Place conceptual indicators and the Aging in the Right Place-Hospice Environmental Assessment Protocol tool therapeutic goals.**

**Built and natural environment**

- Goal 1: provision of access to outside view and nature
- Goal 2: regulate stimulation and support sensory therapies

**Housing access and home modification**

- Goal 3: continuity of self
- Goal 4: culturally appropriate, relevant, and supportive spiritual care

**Emotional place attachment**

- Goal 5: provision of privacy
- Goal 6: provision of autonomy

**Social support, participation, and inclusion**

- Goal 7: facilitate social interaction
- Goal 8: accommodation for loved ones
- Goal 9: support after death
- Goal 10: maximize support for staff

**Safety and Security**

- Goal 11: maximizing safety and security

### Audit Tool Revision

The AIRP-HEAP audit tool is designed to collect data on the presence or absence of environmental features with the options: “Yes,” “No,” or “N/A.” According to Kader [20], when a majority of these items are present in a hospice setting, it suggests that the therapeutic goals are being achieved. For this study, Kader’s original HEAP tool [20] was revised to better align with the needs of AIRP study participants, as the original tool was initially designed to evaluate hospice sites for the general population. With input from relevant partners, the team, consisting of the principal investigators and master’s students, made several adaptations to Kader’s [20] tool to include considerations for the unique needs of OPEH in hospice. For example, the goal “spiritual care” was reconceptualized as “culturally appropriate, relevant, and supportive spiritual care” to align with the needs of the Indigenous population, who are overrepresented in homeless counts in Metro Vancouver [1] and elsewhere in Canada [23]. In addition, the definition of “family accommodation” was expanded from “family members” to “loved ones” to represent the diverse support networks of OPEH who receive care and support from friends and close nonkin loved ones [24]. To adopt a less biomedical approach, we replaced “patient” and “nurse” with “staff,” “individuals,” and “people.” Furthermore, we added an open-ended comment section for each goal to allow auditors to provide detailed notes for additional context as needed.

While the AIRP-HEAP tool captures data about the indoor space of hospice sites, a separate, complementary tool, the AIRP-HEAP secondary observation tool, is used to capture contextual data on the neighborhood surrounding the hospice site. The AIRP-HEAP secondary observation tool was adapted from the qualitative observation section of the Stakeholders Walkability/Wheelability Audit in Neighborhoods (SWAN) tool [25], which is a user-led audit tool that evaluates the environmental barriers to walkability and wheelability of neighborhoods for diverse older adults and persons with disabilities. The AIRP-HEAP secondary observation tool consists of 6 open-ended questions about land use, area characteristics, quality of public space and amenities, feelings of safety, travelling on foot or wheeling, and overall traffic volume, speed, and noise.

### Data Collection

The 2 trained master‘s-level research assistants will independently audit the hospice sites using the AIRP-HEAP and AIRP-HEAP secondary observation tool. The data collection will take around 2 hours to complete. The audit data will be subsequently exported and organized into a Microsoft Excel file, separated by hospice site for analysis. The raw data will be checked to identify blank entries. If blank entries are identified, they will be discussed with the research assistant who conducted the audit to identify the reason and will be subsequently addressed. Given that 2 research assistants will independently audit each site, raters’ answers will be compared to determine if each response is the same (“=TRUE”) or different (“=FALSE”). The discrepancies will be identified and discussed to understand the reason behind the differences, which will then be resolved by consensus. The final data used for analysis will be a consensus of answers between both research assistants. The “N/A” responses will be omitted from scoring due to their lack of applicability. For instance, if the primary question (eg, room present to accommodate family and loved ones) is answered “No,” then the subquestions (eg, about the size of that room) would be considered not applicable (“N/A”). Qualitative data collected with the AIRP-HEAP secondary observation tool will be imported into NVivo (version 14; Lumivero) [26] for analysis.

### Data Analysis

After a consensus is reached on the presence and absence ratings for each item (“Yes” and “No”), the data will be converted into quantitative scores (Yes=1; No=0). Quantitative data will be summed, and a total score (%) for each subobjective will be calculated. A 5-level Likert scale will be created to rate each design objective and follow a scoring system where 0%-20%=“Unusually poor,” 21%-40%=“Poor,” 41%-60%=“Moderate,” 61%-80%=“Strong,” and 81%-100%=“Exceptional.” An overall goal score will not be calculated, as each goal has differing design objectives and subobjectives, thus having inequivalent denominators, which would result in an unbalanced interpretation of the data. Therefore, scoring and interpretation will be performed based on the design objectives within each therapeutic goal. Using NVivo (version 14), the qualitative AIRP-HEAP secondary observation data will be coded line-by-line and subsequently collated to form conceptual categories. Two or more trained research assistants will code the qualitative data to ensure rigor and reach consensus. This will initiate the development of a codebook for the AIRP-HEAP secondary observation data that will be derived from the AIRP theoretical framework, which consists of the 6 indicators of aging in the right place. Coders will meet regularly with the principal investigator to discuss any discrepancies or challenges that arise during the coding process to come to a consensus. Coders will conclude coding when no new themes arise and thematic saturation has been reached.

This protocol is reported in accordance with the Consolidated Criteria for Reporting Qualitative Research (COREQ) checklist to improve transparency, ensure full alignment with qualitative reporting standards, and facilitate the peer-review process. A completed COREQ checklist is provided in Multimedia Appendix 1, where each criterion has been addressed in the manuscript.

### Significance Score Versus Total Score

Within the AIRP-HEAP tool, certain items were noted as “significant” design objectives based on Kader’s [20] original HEAP tool. The significance of items was determined through literature review, expert consultation, and case evidence [20]. For example, some criteria, such as private rooms, are significant and must achieve an optimal level of environmental quality. These are typically well-established, appearing across all 3 phases of data collection or consistently in case studies. Other criteria serve as optional best practices, noted by a few experts or present in select case studies, and can improve overall scores. Some considerations address building layout (eg, distribution of social spaces), while others concern design quality (eg, use of natural materials like wood and stone). Because each criterion is unique, evaluation patterns vary and depend on the nature of the design feature. To further confirm the significance of these items for aging and end-of-life care in the right place for OPEH, we performed an in-depth review of relevant literature.

In Multimedia Appendix 2, the values under “Significant score” will factor in the weight of these significant scores (as a percentage), while the values under “Total score” will consider all items without accounting for significance (as a percentage). “Significant score” will be calculated by dividing the number of significant objectives that are marked present at the hospice site by the total number of significant objectives as labeled in the HEAP tool. This score will allow us to focus only on those characteristics of the hospice that have broader implications (eg, are mandatory) for aging and end-of-life care in the right place for OPEH. “Total score” will be calculated by dividing the number of objectives that are marked present at the hospice site by the total number of objectives, including the significant objectives. This score will allow us to calculate the overall rating of the hospice site based on the Likert scale, and comparisons between hospice sites will mainly be based on this score. Comparing the “Significant score” and “Total score” provides a comprehensive assessment of the quality of the built environment at the hospice sites.

### Reliability and Validity of the Tool

The HEAP was developed following the conceptual framework of PEAP, which is one of the most widely used environmental evaluation tools. Early conceptual work by Weisman [21] and later scholarship on person-centered environmental assessment [22] positioned the PEAP as a framework for understanding how the built environment influences residents’ well-being and daily function. Its reliability has been demonstrated through strong interrater agreement and consistent scoring across trained evaluators [27,28]. The tool’s validity is well supported. Its content aligns with key therapeutic domains, such as safety, comfort, accessibility, social engagement, and cognitive support, while construct validity has been confirmed by associations between PEAP scores and resident outcomes, including functional performance, agitation reduction, and overall satisfaction [29]. More recently, related research has expanded the field of environmental evaluation, with tools such as the Environmental Audit Screening Evaluation further validating evidence-based design approaches [30] and contemporary reviews emphasizing the enduring value of person-centered frameworks for dementia care [31]. Collectively, these studies confirm that HEAP remains a reliable and valid method for guiding design evaluation and improvement in long-term care and dementia-supportive environments.

### Ethical Considerations

The protocol for AIRP under the title “Aging in the Right Place: Building Capacity for Promising Practices that Support Older People Experiencing Homelessness in Montreal, Calgary, and Vancouver” was approved by the University of British Columbia (UBC) Rise system through the UBC and Simon Fraser University Research Ethics Board at Simon Fraser University (H24-01268), McGill University (20-09-008), and the University of Calgary (REB20-1229) ethics boards. All study sites provided informed consent.

## Results

The results based on the findings will contribute to the understanding of the significance and influence of environmental factors in the end-of-life care of hospice residents with experiences of homelessness. Data collection using the AIRP-HEAP and AIRP-HEAP secondary observation tools was conducted at Maggie’s Lodge between November and December 2024. The collected data are currently being organized and cleaned, with analysis and interpretation expected to take place between May and December 2025. As this protocol focuses on environmental audits rather than participant recruitment, no human subject enrollment data are reported. Ethics approval was obtained prior to data collection.

The analysis is expected to generate several key outputs. Quantitatively, the study will produce detailed environmental feature scores for each of the tool’s therapeutic goals. The AIRP-HEAP’s scoring system will allow comparisons between total scores and significance-weighted scores, helping to identify which design objectives are most critical to resident well-being. These findings will highlight both strengths and gaps in the built environment that influence the quality of end-of-life care for residents with experiences of homelessness. Qualitatively, open-ended observations and field notes captured through the AIRP-HEAP secondary observation tool will provide contextual insights into how neighborhood and site-level factors affect the hospice experience. These observations are expected to reveal environmental facilitators and barriers that shape residents’ sense of autonomy, dignity, and inclusion within the hospice setting. Drawing from these insights, the study will generate recommendations for equitable hospice design, focusing on features that promote comfort, belonging, and person-centered care.

## Discussion

### Overview

This protocol aims to provide a comprehensive methodology for conducting a study focused on assessing hospice settings for OPEH in Metro Vancouver. Through this study, new insights into the environmental aspects that influence OPEH’s ability to age and receive end-of-life care in suitable settings will be gained, ultimately contributing to the well-being of this population. Thus, this study allows us to discover information regarding AIRP concepts related to HEAP goals for end-of-life care and identify the environmental facilitators and barriers to improvements in hospice settings in the neighborhoods.

For capturing data and information on this protocol, the AIRP-HEAP tool (for indoor space) includes 295 items categorized into 11 therapeutic goals pertaining to the built and natural environment of hospice sites, and the AIRP-HEAP secondary observation tool (for surrounding neighborhoods) will be used. The most detailed questions in the AIRP-HEAP tool domains are related to the inside of the building and will be asked through a questionnaire in order to evaluate the quality of end-of-life care space. These tools will be used to assess the indoor built environment and the outdoor environment surrounding Maggie’s Lodge hospice in the Metro Vancouver area. Built environment factors that enhance safety, reduce stress, and have spiritual significance will be audited to evaluate the quality of the built environment. For instance, assessing the amount of natural light in the building, ensuring security and privacy measures, providing spiritual and counseling spaces, and creating areas that facilitate social communication are included in the audit tool. Additionally, elements like accessible and wheelable sidewalks, traffic calming features, sufficient outdoor spaces, support services, and social amenities in the surrounding environment are included in the secondary observation tool, as these factors are known to promote potential for social and outdoor engagement for OPEH and their loved ones.

To ensure that the solutions recognized through this protocol can be extended to other hospice centers, we will develop a set of recommendations based on the findings. These recommendations will outline the best practices and actionable steps for improving the built environment and surrounding neighborhoods of hospice settings catering to OPEH. A recent analysis of Canadian homelessness and palliative policy documents highlighted that there is limited guidance for services to provide end-of-life care for OPEH [32]. Additionally, we will share the results of the study, along with the developed guidelines, with relevant stakeholders, including policy makers, urban planners, architects, and health care providers. By disseminating this information widely and encouraging its implementation in other hospice centers, we can promote the adoption of similar improvements across different locations, thereby extending the benefits to a broader population of OPEH. Findings from this study will be context-specific to the selected hospice site, limiting generalizability. However, the adapted AIRP-HEAP tool and conceptual approach can be transferable and applicable to other hospice contexts. By providing a structured framework for examining spatial and organizational dimensions of hospice environments, the tool may support other sites in identifying ways to create more compassionate and inclusive spaces for OPEH.

### Limitations

This study will be conducted exclusively at Maggie’s Lodge hospice during a specific time frame to evaluate the quality of its built environment and the surroundings of the building. While the findings based on the audit tool are expected to contribute to addressing gaps in the literature, the study will be subject to several limitations. First, as the audit will take place at a single point in time, the data will not capture environmental changes that occur over time. Future studies will be able to examine temporal factors and weather impacts on the outdoor spaces of hospices over time. Additionally, while the AIRP-HEAP tool will offer valuable insights into hospice environments, its adaptation from an existing tool may overlook certain unique needs of OPEH. The study’s focus on Maggie’s Lodge hospice site in Metro Vancouver will limit the generalizability of the findings to a broader geographical and cultural context. Finally, findings may not accurately depict the available resources to the hospice, as it omits staff interviews.

### Conclusions

The development of the AIRP-HEAP and AIRP-HEAP secondary observation audit tools adds diversity and detail to existing hospice environment assessment methods by incorporating the unique needs of OPEH. Defining therapeutic goals and creating a design checklist for each goal establishes a milestone for theory and practice. The findings from these tools will help identify spatial design and placemaking features within hospice environments that either promote or deter end-of-life care for this population. By aligning the AIRP-HEAP goals to AIRP-related concepts, this research is expected to strengthen the connection between design-related therapeutic goals and research-based conceptualizations of aging and end-of-life care in the appropriate setting. The anticipated findings of the audit are expected to provide guidance for identifying environmental improvements in hospice settings, as well as inform future planning and design guidelines for hospice settings that support OPEH.

## Appendix

Multimedia Appendix 1 COREQ checklist.

Multimedia Appendix 2 Example of design scores across 11 therapeutic goals.

## Abbreviations

AIRP: Aging in the Right Place
AIRP-HEAP: Aging in the Right Place-Hospice Environmental Assessment Protocol
COREQ: Consolidated Criteria for Reporting Qualitative Research
HEAP: Hospice Environmental Assessment Protocol
OPEH: older people experiencing homelessness
PEAP: Professional Environmental Assessment Protocol
SWAN: Stakeholders Walkability/Wheelability Audit in Neighborhoods
UBC: University of British Columbia

## References

[1] (2020). 2020 homeless count in Metro Vancouver: for reaching home’s community entity for Greater Vancouver. BC Non-Profit Housing Association.

[2] (2023). 2023 homeless count in Greater Vancouver. Prepared for the Greater Vancouver reaching home community entity. Homelessness Services Association of BC.

[3] Canham S, Weldrick R, Sussman T, Walsh CA, Mahmood A (2022). Aging in the right place: a conceptual framework of indicators for older persons experiencing homelessness. Gerontologist.

[4] Rolfe S, Garnham L, Godwin J, Anderson I, Seaman P, Donaldson C (2020). Housing as a social determinant of health and wellbeing: developing an empirically-informed realist theoretical framework. BMC Public Health.

[5] Canada H (2018). Framework on palliative care in Canada. Government of Canada.

[6] Jones A, Vila-Rodriguez F, Leonova O, Langheimer V, Lang DJ, Barr AM, Procyshyn RM, Smith GN, Schultz K, Buchanan T, Krausz M, Montaner JS, MacEwan G W, Rauscher A, Panenka WJ, Thornton AE, Honer WG (2015). Mortality from treatable illnesses in marginally housed adults: a prospective cohort study. BMJ Open.

[7] Biedrzycki B (2018). Homeless with cancer: an unrecognized problem in the United States. Clin J Oncol Nurs.

[8] Sumalinog R, Harrington K, Dosani N, Hwang SW (2017). Advance care planning, palliative care, and end-of-life care interventions for homeless people: a systematic review. Palliat Med.

[9] Mahmood A, Patille R, Lam E, Mora DJ, Gurung S, Bookmyer G, Weldrick R, Chaudhury H, Canham SL (2022). Aging in the right place for older adults experiencing housing insecurity: an environmental assessment of temporary housing program. Int J Environ Res Public Health.

[10] Hutt E, Whitfield E, Min SJ, Jones J, Weber M, Albright K, Levy C, O'Toole T (2016). Challenges of providing end-of-life care for homeless veterans. Am J Hosp Palliat Care.

[11] Wales J, Kurahashi AM, Husain A (2018). The interaction of socioeconomic status with place of death: a qualitative analysis of physician experiences. BMC Palliat Care.

[12] Stajduhar KI, Mollison A, Giesbrecht M, McNeil R, Pauly B, Reimer-Kirkham S, Dosani N, Wallace B, Showler G, Meagher C, Kvakic K, Gleave D, Teal T, Rose C, Showler C, Rounds K (2019). "Just too busy living in the moment and surviving": barriers to accessing health care for structurally vulnerable populations at end-of-life. BMC Palliat Care.

[13] Giesbrecht M, Stajduhar KI, Mollison A, Pauly B, Reimer-Kirkham S, McNeil R, Wallace B, Dosani N, Rose C (2018). Hospitals, clinics, and palliative care units: Place-based experiences of formal healthcare settings by people experiencing structural vulnerability at the end-of-life. Health Place.

[14] Reimer-Kirkham S, Stajduhar K, Pauly B, Giesbrecht M, Mollison A, McNeil R, Wallace B (2016). Death is a social justice issue: perspectives on equity-informed palliative care. ANS Adv Nurs Sci.

[15] McGann S (2016). The Production of Hospice Space: Conceptualising the Space of Caring and Dying. 1st edition.

[16] Arnup K (2013). Death, dying, and Canadian families. Vanier Institute.

[17] Webb W (2015). When dying at home is not an option: exploration of hostel staff views on palliative care for homeless people. Int J Palliat Nurs.

[18] Canham SL, Humphries J, Sussman T, Walsh CA (2020). ging in the right place: a report of promising practice shelter/housing models for older people experiencing homelessness in Montreal, Calgary, and Vancouver. Simon Fraser University.

[19] Golant SM (2015). Aging in the Right Place.

[20] Kader S (2016). Development of Hospice Environmental Assessment Protocol (HEAP): a post occupancy evaluation tool [dissertation]. University of Kansas.

[21] Weisman G (1994). A tool for assessing SCU environments. Nurs Homes Longterm Manag Care.

[22] Chaudhury H, Cooke HA (2015). Person-centered assessment of the physical environment of care settings. Gerontologist.

[23] (2023). Everyone counts 2020-2022: preliminary highlights report. Government of Canada.

[24] Klop H, de Veer AJE, van Dongen SI, Francke AL, Rietjens JA C, Onwuteaka-Philipsen BD (2018). Palliative care for homeless people: a systematic review of the concerns, care needs and preferences, and the barriers and facilitators for providing palliative care. BMC Palliat Care.

[25] Mahmood A, O’Dea E, Bigonnesse C, Labbe D, Mahal T, Qureshi M, Mortenson WB (2019). Stakeholders Walkability/Wheelability Audit in Neighbourhoods (SWAN): user-led audit and photographic documentation in Canada. Disabil Soc.

[26] (2018). NVivo qualitative data analysis software [Software]. QSR International.

[27] Norris-Baker C, Weisman GD, Lawton MP, Sloane P, Kaup M, Steinfeld E, Danford GS (1999). Assessing special care units for dementia: the professional environmental assessment protocol. Enabling environments: Measuring the Impact of Environment on Disability and Rehabilitation.

[28] Lawton MP, Weisman GD, Sloane P, Norris-Baker C, Calkins M, Zimmerman SI (2000). Professional environmental assessment procedure for special care units for elders with dementing illness and its relationship to the therapeutic environment screening schedule. Alzheimer Dis Assoc Disord.

[29] Slaughter SE, Morgan DG (2012). Functional outcomes of nursing home residents in relation to features of the environment: validity of the professional environmental assessment protocol. J Am Med Dir Assoc.

[30] Kaup M, Calkins MP, Davey A, Wrublowsky R (2023). The environmental audit screening evaluation: establishing reliability and validity of an evidence-based design tool. Innov Aging.

[31] Calkins MP, Kaup ML, Abushousheh AM (2022). Evaluation of environmental assessment tools for settings for individuals living with dementia. Alzheimers Dement (NY).

[32] Walsh JJ, Sussman T, Bosma H, Carter RZ, Canham SL, Cormier (2025). The intersections of palliative care and homelessness in social policy: a content analysis of Canadian policy documents. BMC Palliat Care.

