# Supplementary material for: Creating Compassionate Spaces for End-of-Life Care for Older People Experiencing Homelessness: Protocol for an Environmental Assessment of Hospice Settings
Source: JMIR Res Protoc. 2025 Nov 5;14:e73356. doi: 10.2196/73356 (PMC12631086; doi:10.2196/73356)
Supplement: Multimedia Appendix 2 [file resprot_v14i1e73356_app2.docx]

**Table S1.** Example of design scores across 11 therapeutic goals.

|  | **Score** | | **Rating** | |
| --- | --- | --- | --- | --- |
|  | Significant | Total | Significant | Total |
| **Goal 1: Continuity of Self** |  |  |  |  |
| Objective 1: Non-institutional environment or home-like environment |  |  |  |  |
| Objective 2: Scope for personalization |  |  |  |  |
| **Goal 2: Provision of Access to Outside View/Nature** |  |  |  |  |
| Objective 1: Maximize daylight, views, and fresh air through design |  |  |  |  |
| **Goal 3: Provision of Privacy** |  |  |  |  |
| Objective 1: Provides privacy in individual rooms |  |  |  |  |
| Objective 2: Privacy concerns in social spaces, circulation spaces, and in outdoor areas are attended to |  |  |  |  |
| **Goal 4: Facilitate Social Interaction** |  |  |  |  |
| Objective 1: Opportunities in individual rooms |  |  |  |  |
| Objective 2: Opportunities to interact in social or common spaces |  |  |  |  |
| Objective 3: Opportunities in outdoor social spaces |  |  |  |  |
| **Goal 5: Maximize Safety and Security** |  |  |  |  |
| Objective 1: Mitigation of potential hazards |  |  |  |  |
| Objective 2: Provides support and preparedness for infection control |  |  |  |  |
| Objective 3: Provides support for security from theft and vandalism |  |  |  |  |
| **Goal 6: Provision of Autonomy** |  |  |  |  |
| Objective 1: Control over microenvironment and physical settings |  |  |  |  |
| Objective 2: Control over daily routine and activities |  |  |  |  |
| **Goal 7: Regulate Stimulation and Support Sensory Therapies** |  |  |  |  |
| Objective 1: Provides or enhances positive therapeutic stimuli through environmental design |  |  |  |  |
| Objective 2: Regulates levels of acoustic, visual, and olfactory stimulation |  |  |  |  |
| Objective 3: Provides support for therapies |  |  |  |  |
| **Goal 8: Culturally Appropriate/Relevant/Supportive Spiritual Care** |  |  |  |  |
| Objective 1: Facilitates support of spiritual care and cultural practices |  |  |  |  |
| Objective 2: Provides facilities for spiritual care and cultural sensitivities in formal spaces, informal spaces, and in individual rooms |  |  |  |  |
| **Goal 9: Accommodation for Loved Ones** |  |  |  |  |
| Objective 1: Easy accessibility and wayfinding |  |  |  |  |
| Objective 2: Provides comfortable accommodation for loved ones to stay for a few days or for a short visit |  |  |  |  |
| **Goal 10: Support After Death** |  |  |  |  |
| Objective 1: Provides support during the moment of death |  |  |  |  |
| Objective 2: Provides support for deceased individual’s body removal or transfer from the building |  |  |  |  |
| Objective 3: Provides support after death and departure of deceased individual |  |  |  |  |
| **Goal 11: Maximize Support for Staff** |  |  |  |  |
| Objective 1: Provides privacy and comfort for staff |  |  |  |  |
| Objective 2: Provides support for socialization, relaxation, and recreation |  |  |  |  |
| Objective 3: Provides support to ease in observation and care |  |  |  |  |
